# Physiological dead space and alveolar ventilation in ventilated infants

**DOI:** 10.1038/s41390-021-01388-8

**Published:** 2021-02-18

**Authors:** Emma Williams, Theodore Dassios, Paul Dixon, Anne Greenough

**Affiliations:** 1 Department of Women and Children’s Health, School of Life Course Sciences, Faculty of Life Sciences and Medicine, King’s College London, London, UK; 2 Asthma UK Centre for Allergic Mechanisms in Asthma, King’s College London, London, UK; 3 Neonatal Intensive Care Unit, King’s College Hospital NHS Foundation Trust, London, UK; 4 Individual Consultant, London, UK; 5 NIHR Biomedical Centre at Guy’s and St Thomas NHS Foundation Trust, King’s College London, London, UK

## Abstract

**Background:**

Dead space is the volume not taking part in gas exchange and, if increased, could affect alveolar ventilation if there is too low a delivered volume. We determined if there were differences in dead space and alveolar ventilation in ventilated infants with pulmonary disease or no respiratory morbidity.

**Methods:**

A prospective study of mechanically ventilated infants was undertaken. Expiratory tidal volume and carbon dioxide levels were measured. Volumetric capnograms were constructed to calculate the dead space using the modified Bohr–Enghoff equation. Alveolar ventilation (*V*_A_) was also calculated.

**Results:**

Eighty-one infants with a median (range) gestational age of 28.7 (22.4–41.9) weeks were recruited. The dead space [median (IQR)] was higher in 35 infants with respiratory distress syndrome (RDS) [5.7 (5.1–7.0) ml/kg] and in 26 infants with bronchopulmonary dysplasia (BPD) [6.4 (5.1–7.5) ml/kg] than in 20 term controls with no respiratory disease [3.5 (2.8–4.2) ml/kg, *p* < 0.001]. Minute ventilation was higher in both infants with RDS or BPD compared to the controls. *V*_A_ in infants with RDS or BPD was similar to that of the controls [*p* = 0.54].

**Conclusion:**

Prematurely born infants with pulmonary disease have a higher dead space than term controls, which may influence the optimum level during volume-targeted ventilation.

**Impact:**

- Measurement of the dead space was feasible in ventilated newborn infants.
- The physiological dead space was a significant proportion of the delivered volume in ventilated infants.
- The dead space (per kilogram) was higher in ventilated infants with respiratory distress syndrome or evolving bronchopulmonary dysplasia compared to term controls without respiratory disease.
- The dead space volume should be considered when calculating the most appropriate volume during volume-targeted ventilation.

## Introduction

Newborn infants often require respiratory support with invasive mechanical ventilation, unfortunately such infants can develop chronic respiratory morbidity.^1^ Use of volume-targeted ventilation is a lung protective strategy as it potentially avoids too high or too low delivered volumes.^2^ Delivery of inappropriately large tidal volumes can lead to alveolar over-distension and development of chronic respiratory morbidity.^3^ Inappropriately small tidal volumes can be associated with a prolonged duration of mechanical ventilation, a pro-inflammatory state and an increased work of breathing.^4,5^

An important influence on the appropriate size of the delivered volume during mechanical ventilation is the size of the dead space, the volume of inhaled gas that does not take part in gas exchange. The physiological dead space is the anatomical dead space plus alveolar dead space. The anatomical dead space is the total volume of the conducting airways from the nose or mouth to the terminal bronchioles, and in ventilated infants includes the apparatus dead space (endotracheal tube and flow sensor). Alveolar dead space comprises alveoli which are ventilated, but not supplied by the pulmonary arterial circulation, or alveoli which are atelectatic.^6^ In pulmonary diseases in infants, there are a variety of pathologies and hence the dead space is not predictable and has rarely been reported.^7–9^

Minute ventilation fails to adequately describe ventilation efficiency as it does not differentiate between alveolar (effective) and dead space ventilation. In contrast, knowledge of the alveolar ventilation (the volume of air that reaches the alveoli per minute) provides information on the volume of gas taking part in gas exchange at the alveolar–capillary interface.^10^ A previous study has shown that a reduction in alveolar ventilation resulted in hypercapnia in ventilated infants with respiratory distress syndrome (RDS).^11^

The aim of this study was to determine if the physiological dead space in prematurely born infants with RDS or evolving/established bronchopulmonary dysplasia (BPD) was greater than in term controls with no respiratory disease. We further aimed to determine whether the size of the dead space influenced the alveolar ventilation in mechanically ventilated infants.

## Methods

### Study design and participants

A prospective study of ventilated infants at King’s College Hospital NHS Foundation Trust neonatal intensive care unit was undertaken. Infants were recruited from 1 January 2019–1 September 2020 after written informed parental consent was obtained. Approval was given by the London Camden and King’s Cross Research Ethics Committee (REC reference: 18/LO/1602).

Term and preterm infants were eligible for recruitment into the study if they were receiving invasive mechanical ventilation, but did not have major congenital or chromosomal abnormalities. Infants were supported by volume-targeted or pressure-controlled time-cycled ventilation using the SLE6000 neonatal ventilator (SLE, Croydon UK). Infants were intubated, as per unit policy, with shouldered Cole’s endotracheal tubes (ETT).^12^ All preterm infants who required intubation and ventilation received surfactant.

Term born infants with no underlying respiratory disease who required invasive mechanical ventilatory support for poor perinatal adaptation (term controls), preterm infants with acute RDS, and preterm infants supported by invasive ventilation for longer than 1 week who were classified as having evolving^9^ or established BPD were recruited.

### Protocol

Baseline demographic data were collected including gestational age, birth weight and sex. On the day of study postmenstrual age, postnatal age and current weight were recorded. The average respiratory rate was recorded from the bedside monitor prior to measuring each infant. The partial arterial or capillary pressure of carbon dioxide (PaCO_2_) was documented from the latest blood gas analysis which was routinely performed by the clinical team and where possible corresponded with the timing of the research recordings. To collect the ventilatory and respiratory measurement data a 10-min recording was made using the Capnostat-5 sensor, which was inserted between the ETT and ventilator circuit.

### Respiratory measurements

**Fig. 1:**
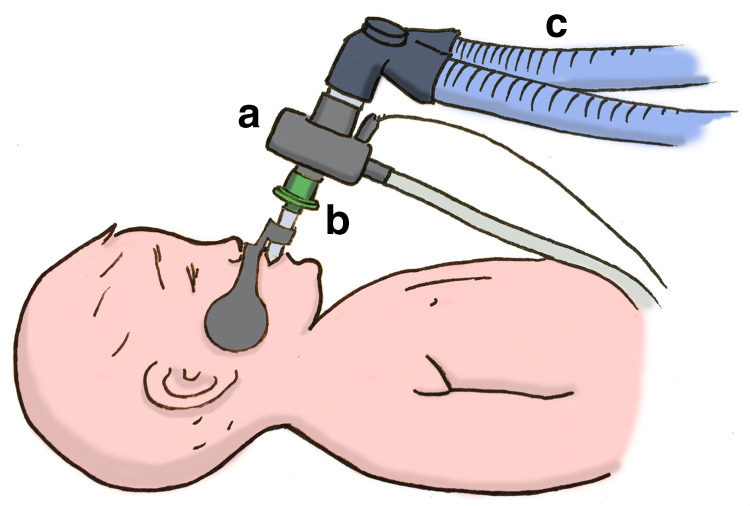
Position of monitoring device within the circuit. **a** combined pneumotachograph and mainstream capnograph **b** Endotracheal tube **c** Ventilator tubing.

An NM3 respiratory profile monitor (Philips Respironics, CT), connected to a combined pressure and flow sensor with mainstream capnograph (Capnostat-5), was used [Fig. 1]. The flow and pressure measurements were from a fixed orifice pneumotachometer and differential pressure sensor, respectively. The expired CO_2_ was measured using infrared absorption spectroscopy. The respiratory monitor was automatically calibrated for pressure, flow and CO_2_ according to the factory-stored calibrations within the monitor. The NM3 monitor was connected to a Laptop (Dell Latitude, UK) with customised Spectra software (3.0.1.4; Grove Medical, UK). The data were exported from Spectra to Microsoft Excel (for Office 365, 2007 version) with a sampling output of 100 Hz for analysis and construction of volumetric capnograms.

### Volumetric capnography

The start of expiration was defined as the start of negative flow with the end of expiration defined as the end of negative flow. There was no delay between the CO_2_ and flow signals with the maximal end-tidal CO_2_ aligning with the end of expiration as determined by the flow. Flow data were used to calculate the expired volume during each expiratory phase where flow rate was the change in volume per unit of time. Integration of the volume (derived from the flow signal) and the corresponding CO_2_ signal was used to calculate the mean CO_2_ of the mixed expired air:

$${{P}}_{{{{\mathrm{emean}}}}}{{{\mathrm{CO}}}}_2 = \frac{{\mathop {\int}\limits_{v = {{{\mathrm{{Vender}}}}}_{{{\mathrm{{insp}}}}}}^{{{{\mathrm{{Vender}}}}}_{{{\mathrm{{xp}}}}}} {P{{{\mathrm{{CO}}}}}_2.{{{\mathrm{d}}}}v} }}{{V_{{{\mathrm{{te}}}}}}}$$

To manually construct the volumetric capnograms for each individual breath, the calculated expired volume was plotted on the *x*-axis against the corresponding partial pressure of exhaled CO_2_ on the *y*-axis.

The waveforms of flow and CO_2_ were deemed to be of sufficient quality for analysis if, after capnogram construction, there were five consecutive breaths of ventilator inflations that were free from artefact interference and allowed for adequate definition between the three phases of the volumetric capnogram. The three phases were related to firstly the emptying of CO_2_ free gas from the conducting airways, secondly the mixing of gas from the conducting airways with CO_2_ rich alveolar gas, and finally during the last phase of expiration which represents pure alveolar CO_2_.^13^ The calculated values for each of the five breaths were averaged for each infant and subsequently used for the dead space calculations.

### Dead space and alveolar ventilation calculations

The dead space was calculated from the Enghoff modification of the Bohr equation by substituting the partial pressure of alveolar carbon dioxide (*P*_A_CO_2_) with arterial partial pressure (P_a_CO_2_).^14^ The physiological dead space (*V*_Dphys_) (ml) was calculated from the following equation:

$${{V}}_{{{{\mathrm{Dphys}}}}} = {{V}}_{{{{\mathrm{te}}}}} \times \left( {{{P}}_{{{\mathrm{a}}}}{{{\mathrm{CO}}}}_2-{{P}}_{{{{\mathrm{emean}}}}}{{{\mathrm{CO}}}}_2} \right)/{{P}}_{{{\mathrm{a}}}}{{{\mathrm{CO}}}}_2$$

The anatomical dead space (*V*_Dana_) (ml) was calculated from the following equation:

$${{V}}_{{{{\mathrm{Dana}}}}} = {{V}}_{{{{\mathrm{te}}}}} \times \left( {{{P}}_{{{{\mathrm{Et}}}}}{{{{\mathrm{{CO}}}}}}_2-{{P}}_{{{{\mathrm{emean}}}}}{{{\mathrm{CO}}}}_2} \right)/{{P}}_{{{{\mathrm{Et}}}}}{{{\mathrm{CO}}}}_2$$

The alveolar dead space (*V*_Dalv_) (ml) was calculated from the following:

$${{V}}_{{{{\mathrm{Dalv}}}}} = {{V}}_{{{{\mathrm{Dphys}}}}} - {{V}}_{{{{{\mathrm{{Dana}}}}}}}$$

where *V*_te_ is the expired tidal volume in ml, *P*_a_CO_2_ is the partial pressure of CO_2_ in the blood in mmHg, *P*_Et_CO_2_ is the maximal end-tidal CO_2_ in mmHg, and *P*_emean_CO_2_ is the mean CO_2_ of the mixed expired air (mmHg).^15^

Minute and alveolar ventilation were also calculated:

$${{{\mathrm{Minute}}}}\,{{{\mathrm{ventilation}}}}\left( {{{{\mathrm{ml/kg/min}}}}} \right) = {{V}}_{{{{\mathrm{te}}}}} \times {{{\mathrm{frequency}}}}$$

$${{{\mathrm{Alveolar}}}}\,{{{\mathrm{ventilation}}}}\left( {{{{\mathrm{ml}}}}/{{{\mathrm{kg}}}}/{{{\mathrm{min}}}}} \right) = \left( {{{V}}_{{{{\mathrm{te}}}}} - {{V}}_{{{{\mathrm{Dphys}}}}}} \right) \times {{{\mathrm{frequency}}}}$$

where *V*_te_ is the expired tidal volume in ml/kg, *V*_Dphys_ is the physiological dead space in ml/kg and frequency is the respiratory rate in breaths per minute.

### Apparatus dead space

The apparatus dead space consisted of the ETT and mainstream capnograph with the combined flow and CO_2_ sensor (Capnostat-5). The Capnostat-5 had a dead space volume of 0.8 ml as measured by water displacement and confirmed by the manufacturer. The ETT “dead space” was also measured using the water displacement technique and was 1.2 ml for a size 2.0 mm ETT, 1.8 ml for 2.5 mm ETT, 2 ml for 3.0 mm ETT, 3 ml for 3.5 mm ETT and 3.2 ml for 4.0 mm ETT.

### Sample size calculation

Previous studies have reported a difference in the physiological dead space of 0.8 ml/kg between preterm and term infants^16^ with a standard deviation of the physiological dead space in preterm infants of 0.78 ml.^8^ Therefore, to detect a difference in the physiological dead space of 0.8 ml/kg with 90% power at the 5% significance level a minimum of 20 infants within each group was required. Infants were recruited consecutively into the study until the minimum size in each group had been achieved.

### Statistical analysis

The data were tested for normality using the Kolmogorov–Smirnov test and found to be non-normally distributed. Data are therefore presented as median values with ranges or interquartile ranges (range or IQRs). The Kruskall–Wallis test was used to assess if there were significant differences in the dead space and alveolar ventilation between the three groups, and if significant differences were identified Mann Whitney *U* tests were performed to determine between which groups the differences occurred. Statistical analysis was performed using the SPSS software version 25.0 (SPSS Inc., Chicago IL).

## Results

Eighty-one infants were recruited into the study with a median (range) gestational age of 28.7 (22.4–41.9) weeks and a birth weight of 1.0 (0.43–4.6) kg. Twenty term infants had no underlying lung pathology (controls), 35 preterm infants had RDS and 26 preterm infants had evolving/established BPD. Two infants subsequently needed a tracheostomy and 21 infants developed severe BPD. The control infants were studied at a median (interquartile range) postnatal age of 4 (2–7) days, infants with RDS at 3 (2–4) days and infants with BPD at 16 (10–25) days [Table 1]. At the time of study, the median (IQR) FiO_2_ in the RDS group was 0.24 (0.21–0.30) and in the BPD group was 0.34 (0.26–0.49) (*p* = 0.01).

**Table 1:** Baseline demographics of infants in each group.

	Control (*n* = 20)	RDS (*n* = 35)	BPD (*n* = 26)	*p* value
Gestational age at birth (weeks)	38.4 (36.7–40.2)	29.1 (25.9–31.7)	25.8 (25.0–27.3)	<0.001
Birth weight (kg)	3.30 (2.75–3.66)	1.00 (0.74–1.41)	0.70 (0.58–0.82)	<0.001
BW *Z*-score	−0.07 (−0.72 to 0.63)	−0.41 (−1.56 to 0.00)	−0.76 (−1.66 to −0.19)	0.066
Postnatal day of life at study (days)	4 (2–7)	3 (2–4)	16 (10–25)	<0.001
Postmenstrual age at study (weeks)	39.8 (37.6–40.9)	29.6 (26.1–31.9)	28.3 (26.9–30.2)	<0.001
Weight at study (kg)	3.36 (2.85–3.78)	1.03 (0.74–1.44)	0.91 (0.77–1.18)	<0.001
End-tidal carbon dioxide (mmHg)	31.3 (28.3–39.9)	29.3 (24.2–33.4)	33.7 (28.2–38.4)	0.030
Mean expired carbon dioxide (mmHg)	18.2 (15.4–21.2)	10.6 (7.4–12.7)	11.8 (9.9–15.1)	<0.001
Partial arterial pressure of carbon dioxide (mmHg)	40.4 (34.0–43.1)	40.0 (34.5–43.7)	51.4 (47.8–59.0)	<0.001
PaCO_2_–EtCO_2_ gradient (mmHg)	5.2 (2.8–12.2)	10.0 (5.5–16.3)	17.9 (14.8–27.0)	<0.001
Peak inspiratory pressure (cmH_2_O)	18.7 (16.5–21.5)	16.9 (13.1–20.3)	23.8 (18.8–25.6)	0.006
Positive end expiratory pressure (cmH_2_O)	5.9 (5.4–6.0)	5.4 (5.1–5.9)	6.1 (5.4–6.7)	0.005
Respiratory rate (breaths per minute)	35 (31–39)	45 (40–58)	49 (42–55)	<0.001

Data presented as median with interquartile range (IQR) in brackets.

The control infants had expired tidal volumes of 6.4 (5.4–7.6) ml/kg; which were lower than the expired tidal volumes of infants with RDS [8.5 (6.6–9.6) ml/kg] or BPD [8.2 (7.5–10.3) ml/kg] (*p* = 0.009). Respiratory rate was 35 (31–39) bpm in the term infants and was higher in preterm infants with RDS [45 (40–58) bpm] or BPD [49 (42–55) bpm; *p* < 0.001]. Term infants had similar PaCO_2_ levels [40.4 (34.0–43.1) mmHg] to infants with RDS [40.0 (34.5–43.7) mmHg; *p* = 0.997], but infants with BPD had higher *P*aCO_2_ levels [51.4 (47.8–59.0) mmHg; *p* < 0.001] [Table 1].

**Fig. 2:**
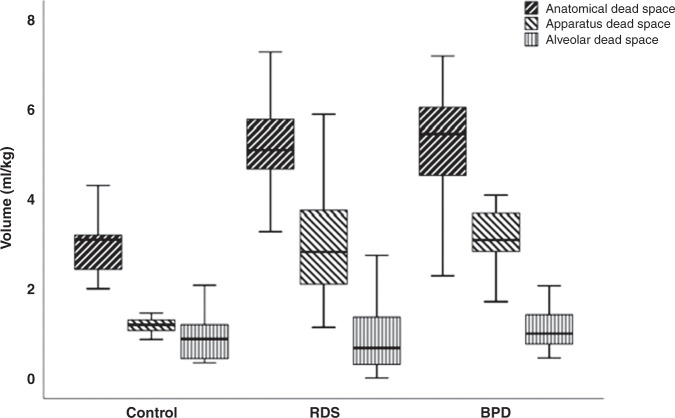
Box and whisker plot of dead space volumes within the three groups. The physiological, anatomical and apparatus dead space contributions are shown for term controls and preterm infants with respiratory distress syndrome and evolving bronchopulmonary dysplasia. The ends of each box are the upper and lower quartiles, with the median marked by a horizontal line inside the box. The whiskers mark the minimum and maximum dead space values.

The physiological dead space was higher in infants with RDS [5.7 (5.1–7.0) ml/kg] or in those with evolving/established BPD [6.4 (5.1–7.5) ml/kg] than in term controls [3.5 (2.8–4.3) ml/kg] (<0.001) [Fig. 2]. There was no significant difference (*p* = 0.37) in the physiological dead space between infants with RDS or BPD. Within the BPD group, there was no significant difference in the physiological dead space in those who survived to discharge versus those who died before discharge (*p* = 0.915). There were also no significant differences in the physiological dead space between infants with mild, moderate or severe BPD (*p* = 0.187). In the controls, the alveolar dead space was 0.49 (0.30–1.16) ml/kg which was lower than in infants with BPD [0.99 (0.75–1.46) ml/kg; *p* = 0.006], but similar to infants with RDS [0.68 (0.24–1.46) ml/kg; *p* = 0.506]. Alveolar dead space was higher in infants with BPD compared to infants with RDS (*p* = 0.03) [Table 2].

**Table 2:** Dead space and alveolar ventilation parameter of infants in each group.

	Control (*n* = 20)	RDS (*n* = 35)	BPD (*n* = 26)	*p* value	Control–RDS	Control–BPD	RDS–BPD
Physiological dead space (ml/kg)	3.5 (2.8–4.3)	5.7 (5.1–7.0)	6.4 (5.1–7.5)	<0.001	<0.001	<0.001	0.372
Total anatomical dead space (ml/kg)	2.7 (2.4–3.2)	5.1 (4.7–5.9)	5.4 (4.4–6.1)	<0.001	<0.001	<0.001	0.811
Apparatus dead space (ml/kg)	1.2 (1.1–1.4)	2.8 (2.0–3.8)	3.1 (2.8–3.7)	<0.001	<0.001	<0.001	0.353
Anatomical minus apparatus dead space (ml/kg)	1.6 (1.1–2.2)	2.4 (1.5–3.2)	2.0 (1.4–2.9)	0.171			
Alveolar dead space (ml/kg)	0.5 (0.3–1.2)	0.7 (0.2–1.5)	1.0 (0.8–1.4)	0.018	0.506	0.006	0.030
Tidal volume (ml/kg)	6.4 (5.4–7.6)	8.5 (6.6–9.6)	8.2 (7.5–10.3)	0.009	0.008	0.004	0.745
Dead space to tidal volume ratio	0.52 (0.43–0.62)	0.75 (0.64–0.81)	0.77 (0.71–0.82)	<0.001	<0.001	<0.001	0.220
Minute ventilation (ml/kg/min)	239 (193–378)	369 (311–450)	403 (321–505)	0.003	0.002	0.001	0.580
Alveolar ventilation (ml/kg/min)	111 (81–159)	83 (66–137)	82 (62–133)	0.537			

Data presented as median with interquartile range (IQR) in brackets.

The total anatomical dead space in term infants was 2.7 (2.8–4.3) ml/kg, which was lower than in infants with RDS [5.1 (4.7–5.9) ml/kg; *p* < 0.001] and infants with BPD [5.4 (4.4–6.1) ml/kg; *p* < 0.001]. The apparatus dead space was lower in term infants [1.2 (1.1–1.4) ml/kg] than preterm infants with either RDS [2.8 (2.0–3.8) ml/kg; *p* < 0.001] or BPD [3.1 (2.8–3.7); *p* < 0.001]. After subtracting the apparatus contribution from the total anatomical dead space there was no significant difference in the remaining anatomical dead space between the three groups (*p* = 0.17) [Table 2].

Minute ventilation was higher in both infants with RDS [369 (311–450) ml/kg/min] or BPD [403 (321–505) ml/kg/min] compared to the controls [239 (193–378) ml/kg/min; *p* = 0.003]. Alveolar ventilation in preterm infants with RDS [83 (66–137) ml/kg/min] or BPD [83 (62–133) ml/kg/min] was not significantly different to that of the term controls [111 (81–159) ml/kg/min; *p* = 0.54] [Table 2].

## Discussion

We have demonstrated that in ventilated newborns, preterm infants with RDS or evolving/established BPD had a higher physiological dead space than term infants without respiratory disease. Furthermore, there was an increase in minute ventilation in preterm infants with underlying lung pathology and as a consequence the alveolar ventilation was similar in the three groups.

The increase in alveolar dead space in infants with evolving/established BPD arises from the underlying disease process. BPD is a heterogeneous lung disease characterised by simplified alveoli with areas of cystic change.^17^ Computerised tomography studies have correlated the severity of BPD with pulmonary hyper-expansion, emphysematous changes and interstitial abnormalities seen on imaging.^18^ Cystic areas developing within the lung parenchyma in infants with evolving/established BPD may explain therefore our findings of an increase in alveolar dead space in comparison to healthy term controls. The BPD and RDS patients had similar V/A, yet the BPD patients had higher pCO_2_ levels. We suggest the mechanism underlying this finding is that infants with BPD develop non-functional cystic areas which do not contribute to gas exchange and clearance of carbon dioxide.

The preterm infants with either RDS or BPD had a greater apparatus dead space volume per kilogram. This finding emphasises the concerns regarding the contribution of the apparatus dead space (ETT and flow sensor) in extremely low birth weight infants who have small tidal volumes.^9,19^

The values of physiological dead space in our study are higher than previously reported.^8,20^ One explanation is that infants in our study were less mature and of lower birth weight than those previously reported and were requiring invasive ventilatory support for underlying lung disease. Furthermore, used the Enghoff modification of the Bohr equation to calculate the dead space—this modification substitutes alveolar CO_2_ with arterial CO_2_ due to the technical and methodological difficulties in obtaining accurate alveolar CO_2_ values. The Bohr equation gives the true dead space, whereas the Enghoff modification may overestimate the dead space as it reflects not only the dead space, but also the degree of intrapulmonary shunt, diffusion impairment and ventilation-perfusion inhomogeneity.^21^

Our study has strengths and some limitations. The European Respiratory Society/American Thoracic Society (ERS/ATS) task force standards for infant respiratory function testing state that the additional dead space of the measuring apparatus should be not more than 2 ml/kg.^22^ We used a combined pressure and flow sensor with mainstream capnograph to calculate the physiological measurements which had a dead space below that recommended for all infants recruited.^22^ A further strength is that we have used the Bohr–Enghoff equation to calculate dead space parameters as opposed to Fletcher’s method, which uses Fowler’s equal triangles to calculate the anatomical dead space and may prove not-applicable in low birth weight infants with high respiratory rates and short expiratory times.^15^ The former method has previously been shown to be superior in ventilated preterm infants and in one study could be used for all infant dead space calculations irrespective of the capnogram shape.^20^ In that study^20^ the single breath CO_2_ test was used which has been superseded by low dead space, fast response CO_2_ analysers which were employed in the present study. Our method of volumetric capnography, however, does require infants to be invasively ventilated and thus is not generalisable to the whole population of preterm infants. Knowledge of the physiological dead space is most relevant in ventilated infants to determine the optimum delivered volume. A further limitation is that we use shouldered endotracheal tubes which are not routinely employed in other units, it would, therefore, be interesting to repeat our study in a unit which uses straight endotracheal tubes. We do not have the exact timings of blood gas analysis, but blood gases were done on a clinical basis and the research measurements were performed as close in time as possible, usually within two hours.

Our study has important clinical implications suggesting that the dead space should be considered alongside other clinical parameters when deciding the magnitude of targeted tidal volumes required to maintain effective alveolar ventilation. Our results suggest that premature infants with RDS or evolving BPD might need higher tidal volume targeting levels compared to term infants with no respiratory disease. It would be interesting to assess whether these early measures of physiological dead space could predict longer term outcomes, this was not possible in this cohort as all but one infant with evolving BPD went on to develop established BPD.

In conclusion we have reported that the physiological dead space is higher in ventilated preterm infants with RDS or evolving/established BPD compared to term controls without respiratory disease. These findings should be taken into consideration when determining the magnitude of the volumes that should be delivered in volume-targeted modes of ventilation.
