# Correction to: Physiological dead space and alveolar ventilation in ventilated infants

**DOI:** 10.1038/s41390-021-01676-3

**Published:** 2021-08-17

**Authors:** Emma Williams, Theodore Dassios, Paul Dixon, Anne Greenough

**Affiliations:** 1 Department of Women and Children’s Health, School of Life Course Sciences, Faculty of Life Sciences and Medicine, King’s College London, London, UK; 2 Asthma UK Centre for Allergic Mechanisms in Asthma, King’s College London, London, UK; 3 Neonatal Intensive Care Unit, King’s College Hospital NHS Foundation Trust, London, UK; 4 Individual Consultant, London, UK; 5 NIHR Biomedical Centre at Guy’s and St Thomas NHS Foundation Trust, King’s College London, London, UK

Correction to: *Pediatric Research* 10.1038/s41390-021-01388-8, published online 18 February 2021

The article Physiological dead space and alveolar ventilation in ventilated infants, written by Emma Williams, Theodore Dassios, Paul Dixon, and Anne Greenough, was originally published electronically on the publisher’s internet portal on 18 February 2021 without open access. With the author(s)’ decision to opt for Open Choice, the copyright of the article changed on 23 June 2021 to © The Author(s) 2021 and the article is forthwith distributed under a Creative Commons Attribution 4.0 International License, which permits use, sharing, adaptation, distribution, and reproduction in any medium or format, as long as you give appropriate credit to the original author(s) and the source, provide a link to the Creative Commons licence, and indicate if changes were made.

The images or other third-party material in this article are included in the article’s Creative Commons licence, unless indicated otherwise in a credit line to the material. If material is not included in the article’s Creative Commons licence and your intended use is not permitted by statutory regulation or exceeds the permitted use, you will need to obtain permission directly from the copyright holder.

To view a copy of this licence, visit http://creativecommons.org/licenses/by/4.0/.

